# Coenzyme Q10 prevents RANKL-induced osteoclastogenesis by promoting autophagy via inactivation of the PI3K/AKT/mTOR and MAPK pathways

**DOI:** 10.1590/1414-431X2024e13474

**Published:** 2024-05-03

**Authors:** Delu Zheng, Chenli Cui, Chengsong Ye, Chen Shao, Xiujing Zha, Ying Xu, Xu Liu, Can Wang

**Affiliations:** 1 Department of Endocrinology, The Second Affiliated Hospital of Bengbu Medical University, Bengbu, Anhui, China; 2 Hefei Institute of Technology Innovation Engineering, Chinese Academy of Sciences, Hefei, Anhui, China; 3 The Operative Surgery Laboratory, Bengbu Medical University, Bengbu, Anhui, China; 4 School of Electronic and Electrical Engineering, Bengbu University, Bengbu, Anhui, China; 5 National Engineering Research Center of Coal Mine Water Hazard Controlling, Suzhou University, Suzhou, Jiangsu, China; 6 School of Earth and Space Sciences, University of Science and Technology of China, Hefei, Anhui, China

**Keywords:** Osteoclastogenesis, Coenzyme Q10, Autophagy, PI3K/AKT/mTOR, MAPK

## Abstract

Coenzyme Q10 (CoQ10) is a potent antioxidant that is implicated in the inhibition of osteoclastogenesis, but the underlying mechanism has not been determined. We explored the underlying molecular mechanisms involved in this process. RAW264.7 cells received receptor activator of NF-κB ligand (RANKL) and CoQ10, after which the differentiation and viability of osteoclasts were assessed. After the cells were treated with CoQ10 and/or H_2_O_2_ and RANKL, the levels of reactive oxygen species (ROS) and proteins involved in the PI3K/AKT/mTOR and MAPK pathways and autophagy were tested. Moreover, after the cells were pretreated with or without inhibitors of the two pathways or with the mitophagy agonist, the levels of autophagy-related proteins and osteoclast markers were measured. CoQ10 significantly decreased the number of TRAP-positive cells and the level of ROS but had no significant impact on cell viability. The relative phosphorylation levels of PI3K, AKT, mTOR, ERK, and p38 were significantly reduced, but the levels of FOXO3/LC3/Beclin1 were significantly augmented. Moreover, the levels of FOXO3/LC3/Beclin1 were significantly increased by the inhibitors and mitophagy agonist, while the levels of osteoclast markers showed the opposite results. Our data showed that CoQ10 prevented RANKL-induced osteoclastogenesis by promoting autophagy via inactivation of the PI3K/AKT/mTOR and MAPK pathways in RAW264.7 cells.

## Introduction

Postmenopausal osteoporosis is an age-related, silent systemic disease characterized by progressive bone mass loss and a greater incidence of bone fracture, mainly due to a marked reduction in estrogen levels after menopause (1). It is a well-known and increasingly common public health issue that contributes to the reduction of well-being and quality of life (2). The number of adults with osteoporosis is estimated to increase to 71 million by 2030 (3). During perimenopause and postmenopause, estrogen deficiency increases oxidative stress and mitochondrial dysfunction (4). Menopausal hormone therapy (MHT) can prevent the damage to mitochondrial function caused by oxidative stress, but long-term MHT increases the risk of cardiovascular and cerebrovascular disorders, stroke, and cancer (5). Current medications for osteoporosis, including bisphosphonates (BPs), have side effects, such as esophageal erosions and ulcers, arthralgia, and renal impairment (6). Thus, it is necessary to discover a potent endogenous antioxidant for postmenopausal osteoporosis.

Coenzyme Q10 (CoQ10) is a redox component of the respiratory chain that contributes to the regulation of energy metabolism and cell death (7,8). In addition, CoQ10 has been confirmed to be the only endogenous antioxidant that inhibits lipid peroxidation and provides protection against oxidative stress injury to mitochondrial proteins and DNA (9). Proteins in subcellular membranes can be uncoupled by CoQ10 as a cofactor, but its main role is to scavenge reactive oxygen species (ROS) from mitochondria and other biological membranes and to act as an antioxidant. Recently, a growing body of evidence has suggested that CoQ10 can concurrently escalate osteoblastogenesis and reduce osteoclastogenesis (10). Our previous studies revealed that the antioxidant CoQ10 could repress osteoclastogenesis induced by receptor activator of NF-κB ligand (RANKL) by modulating mitochondrial apoptosis and oxidative stress (11). However, the underlying mechanism(s) still need to be further clarified.

Autophagy is a preserved catabolic procedure in which cytoplasmic constituents and organelles in the lysosome are degraded (12,13). It is essential for the maintenance of cell homeostasis and stress responses. Multiple autophagic activity-related proteins are important for the growth, death, and differentiation of bone cells, which include osteoclasts (14). Dysregulated levels of autophagic activity interrupt the stability of bone formation and resorption, mediating the initiation and development of a number of bone diseases, including osteoporosis (14,15). Thus, targeting autophagy might be a potentially effective treatment for osteoporosis.

Previous studies confirmed that CoQ10 could alleviate many disorders by regulating autophagy. For example, pretreatment with CoQ10 could decrease myocardial apoptosis and improve cardiac function in an animal model of acute ischemia-reperfusion injury by enhancing autophagy (16). In addition, CoQ10 supplementation protected against liver and lung fibrosis in methotrexate (MTX)-treated rats by upregulating the autophagy pathway (17). Moreover, CoQ10 preconditioning could decrease BPA-induced apoptosis in C2C12 mouse myoblasts via promotion of autophagy (18). However, little information is available about CoQ10 regulating autophagy in osteoporosis.

This study, therefore, aimed to discover the functions of CoQ10 in autophagy during osteoclastogenesis, as well as the potential signaling pathways involved. Our research may provide insights into novel therapies for postmenopausal osteoporosis.

## Material and Methods

### Cell culture

RAW264.7 cells (Wuhan Procell Biological Technology Co., China) were cultured in DMEM (Servicebio, China) supplemented with fetal bovine serum (FBS; Every Green, China) and 2 mM glutamine (Every Green) in a humidified atmosphere of 5% CO_2_ at 37°C.

### Cell differentiation and treatment

To promote differentiation into osteoclasts, RAW264.7 cells were treated with RANKL (Sigma-Aldrich, USA) for six days. After treatment with 50 ng/mL RANKL, CoQ10 (10^-3^ M, Aladdin Reagent Co., Ltd., China) with or without 10^-4^ M H_2_O_2_ was added to the cell line (11). These cells were preconditioned with the inhibitors PI3K (LY294002, 30 μM; #L832989; Macklin, China) for 1 h (19), ERK (PD98059, 10 μM; MedChem Express, USA) for 1 h (20), p38MAPK (SB203580, 10 μM; Yuanye Bio-Technology Co., Ltd., China) for 1 h (21), or mitophagy agonist Torin1 (4 nM; #T861013; Macklin) for half an hour.

### Cell viability assay

MTT assay was performed to test cell viability. Briefly, the cells were seeded in 96-well plates with 5 multiple wells (5×10^3^ cells/well). After treatment, MTT solution (20 μL, #WLA021, Wanlei Bio, China) was added to the wells, which were subsequently incubated at 37°C for approximately 4 h. The formazan crystals were dissolved in 150 μL of dimethyl sulfoxide (DMSO) in the supernatant. MTT values were then determined using a microplate reader (CLARIOstar, BMG LABTECH Inc., USA) at 570 nm.

### ROS testing

2,7-Dichlorofluorescein diacetate (DCFH-DA) (#WLA131; Wanlei Bio) was used to test the intracellular levels of ROS, which included hydroxyl free radicals (radical · OH), hydrogen peroxide (H_2_O_2_), and superoxide anions (O_2_·^−^). CoQ10 was administered to cells with or without 10^-4^ M H_2_O_2_ and treated with 50 ng/mL RANKL. Afterwards, 10 μM DCFH-DA was added to each well, and the mixture was incubated in the dark for 15 min at 37°C. A multifunctional microplate analyzer (Tecan, Infinite M200 Pro, Switzerland) was used to measure the fluorescence values.

### TRAP

After the cells were administered CoQ10 with or without 10^-4^ M H_2_O_2_ and treated with RANKL (50 ng/mL), the cells were plated onto 24-well plates and incubated at 37°C with 5% CO_2_. Subsequently, the medium was removed, and phosphate-buffered saline (PBS; Cat# B548117; Sangon Biotech, China) was used to wash the cells three times. Next, 4% paraformaldehyde was used to fix the cells, after which resistant acid phosphatase (TRAP) staining solution (#D023-1-1; Jiancheng, China) was added to the cells, which were incubated at 37°C away from light for 1 h. The TRAP-positive cells were visualized and quantified using an inverted light microscope (Nikon Eclipse TS100, Japan).

### Western blot

A protein extraction kit (#WLA019) was used to extract total protein, and a BCA quantification kit (#WLA004) was used to determine the concentration of the protein. Both kits were purchased from Wanleibio (China). The samples (20 μL) were subjected to SDS-PAGE and were subsequently transferred to PVDF membranes (#IPVH00010; Millipore, USA). Afterwards, the samples were washed three times with TBST and blocked with bovine serum albumin (BSA, #WLA066; Wanleibio). Later, the membranes were incubated with the following primary antibodies at 4°C overnight: p-PI3K p85 (Tyr458) (#AF3242), PI3K p85 (WL02240), p-AKT (Ser473) (#WLP001a), AKT (#WL0003b), p-mTOR (Ser2448) (#WL03694), mTOR (#WL02477), p-ERK1/2 (thr202/tyr204) (#WLP1512), ERK1/2 (#WL01864), p-P38 (Thr180/Tyr182) (#WLP1576), P38 (#WL00764), FOXO3 (#WL02891), Beclin 1 (#WL02508), LC3-I/II (#WL01506), TRAP (#WL02846), NFATc1 (#WL01632), and OSCAR (#PA5-47171). β-actin (#WL01372) was used as a loading reference. The p-PI3K p85 (Tyr458) and OSCAR antibodies were purchased from Affinity Biosciences (USA) and Thermo Fisher Scientific (USA), respectively. The remaining primary antibodies were obtained from Wanleibio. The membranes were incubated with donkey anti-goat IgG (#A0181; Beyotime Institute of Biotechnology, China) or goat anti-rabbit HRP (#WLA023; Wanleibio) at 37°C for one hour. The band intensity was determined via enhanced chemiluminescence (ECL) and calculated with Gel-Pro-Analyzer software (http://gelanalyzer.com).

### Statistical analysis

The data are reported as means±SD and were analyzed with GraphPad Prism 8.0 (GraphPad Software, Inc., USA). Differences between 2 groups were tested with Student's *t*-test and between 3 or more groups, with one-way analysis of variance (ANOVA). In addition, two-way ANOVA was carried out for comparisons of two independent variables. A P-value <0.05 was considered to indicate a significant difference.

## Results

### CoQ10 inhibited RANKL-induced osteoclastogenesis

**Figure 1:**
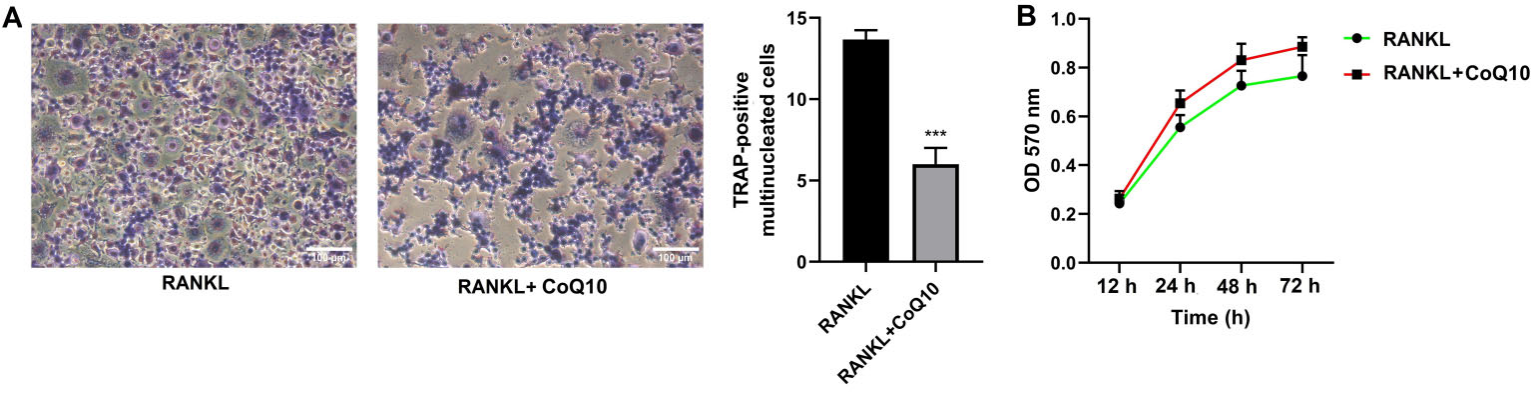
CoQ10 prevents RANKL-induced osteoclastogenesis. After RAW264.7 cells were treated with 50 ng/mL RANKL and 10^-3^ M CoQ10, the differentiation and viability of the osteoclasts were evaluated. **A**, TRAP staining of RAW264.7 cells after treatment with RANKL and CoQ10 (scale bar 100 μm); **B**, Viability of RAW264.7 cells after treatment with RANKL and CoQ10. The data are reported as means±SD. ***P<0.001; *t*-test and ANOVA. CoQ10: Coenzyme Q10; RANKL: receptor activator of NF‐κB ligand; TRAP: tartrate-resistant acid phosphatase.

RANKL is an important regulator of osteoclastogenesis. On the basis of our previous study, we confirmed that the optimal concentration of RANKL for promoting osteoclastogenesis was 50 ng/mL (11). To further determine the effects of CoQ10 on osteoclastogenesis, 10^-3^ M CoQ10 was administered, after which TRAP staining and MTT assays were performed. As reported in Figure 1A, the number of TRAP-positive cells was significantly lower in the RANKL + 10^-3^ M CoQ10 group than in the RANKL group (P<0.001). However, cell viability did not obviously change between the two groups, indicating that 10^-3^ M CoQ10 was nontoxic to the cells (Figure 1B). These data revealed that CoQ10 prevented RANKL-induced osteoclastogenesis.

### CoQ10 inhibited ROS production in RAW264.7 cells

**Figure 2:**
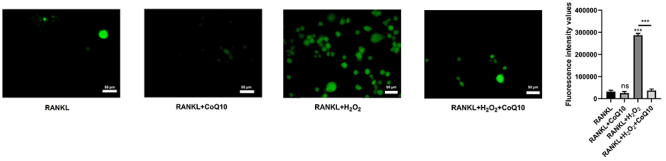
CoQ10 constrains ROS generation in RANKL-treated RAW264.7 cells. DCFH-DA was used to determine the intracellular ROS level after RAW264.7 cells were treated with 10^-3^ M CoQ10 with or without 10^-4^ M H_2_O_2_ in the presence of 50 ng/mL RANKL (scale bar 50 μm). The data are reported as means±SD. ***P<0.001 compared to the control (RANKL), unless otherwise indicated; ANOVA; ns: not significant. CoQ10: Coenzyme Q10; ROS: reactive oxygen species; RANKL: receptor activator of NF‐κB ligand; DCFH-DA: 2,7-dichlorofluorescein diacetate.

Next, we assessed the impact of CoQ10 on ROS production. ROS are primarily produced as a result of H_2_O_2_ entering the membrane structure of biological cells. Therefore, H_2_O_2_ was added as a positive control. Although the relative fluorescence values did not significantly change between the RANKL + 10^-3^ M CoQ10 and RANKL groups, the relative fluorescence values were significantly greater in the RANKL + H_2_O_2_ group but lower in the RANKL + H_2_O_2_ + 10^-3^ M CoQ10 group than in the RANKL + H_2_O_2_ group (P<0.001; Figure 2). These findings suggested that CoQ10 inhibited ROS production in RAW264.7 cells treated with RANKL under oxidative stress.

### CoQ10 promoted autophagy in RAW264.7 cells

**Figure 3:**
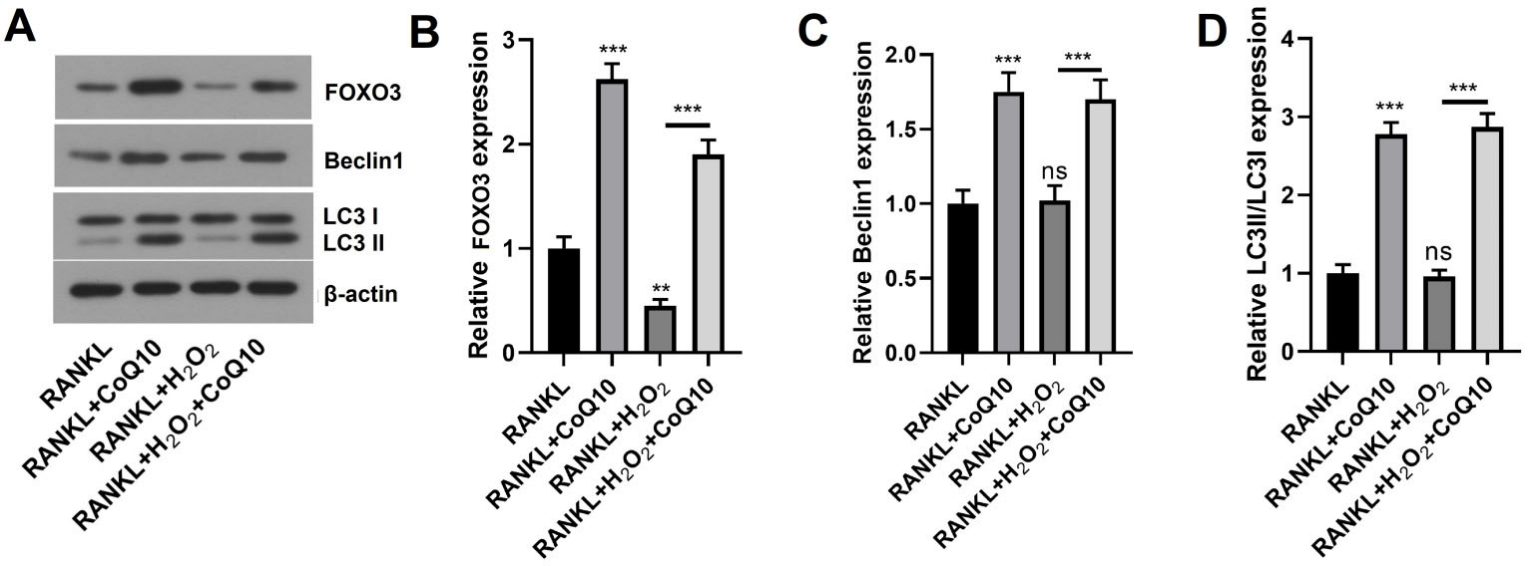
CoQ10 promotes autophagy in RANKL-treated RAW264.7 cells. After the RAW264.7 cells were treated with 10^-3^ M CoQ10 with or without 10^-4^ M H_2_O_2_ in the presence of 50 ng/mL RANKL, the expression of autophagy-related proteins was determined. **A**, Images of autophagy-related proteins determined by Western blot; **B**, Quantitative analysis of FOXO3; **C**, Quantitative analysis of Beclin1; **D**, Quantitative analysis of LC3II/LC3I. CoQ10: Coenzyme Q10; RANKL: receptor activator of NF‐κB ligand; FOXO3: forkhead box protein O3. The data are reported as means±SD. **P<0.01, ***P<0.001 compared to the control (RANKL), unless otherwise indicated; ANOVA; ns: not significant.

Next, we tested the impact of CoQ10 on autophagy-related proteins. Similarly, the relative expression levels of FOXO3, Beclin1, and LC3II/LC3I (all P<0.001) were considerably greater in the RANKL + CoQ10 group than in the RANKL group. Although the relative levels of FOXO3 (P<0.01) were significantly lower in the RANKL + H_2_O_2_ group than in the RANKL group, non-significant differences in the levels of Beclin1 and the LC3II/LC3I were detected between the two groups. In addition, we found that the relative expression levels of FOXO3, Beclin1 and LC3II/LC3I (all P<0.001) were significantly greater in the RANKL + H_2_O_2_ + CoQ10 group than in the RANKL + H_2_O_2_ group (Figure 3A-D). These data suggested that CoQ10 could promote autophagy in RAW264.7 cells treated with RANKL under conditions of oxidative stress.

### CoQ10 inactivated the PI3K/AKT/mTOR and MAPK pathways in RAW264.7 cells

**Figure 4:**
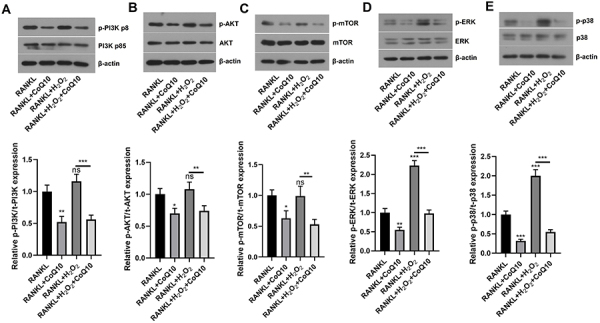
CoQ10 inactivates the PI3K/AKT/mTOR and MAPK signaling pathways in RANKL-treated RAW264.7 cells. After RAW264.7 cells were treated with 10^-3^ M CoQ10 with or without 10^-4^ M H_2_O_2_ in the presence of 50 ng/mL RANKL, PI3K/AKT/mTOR, and MAPK signaling pathway-related proteins were tested. **A**, The levels of p-PI3K/t-PI3K; **B**, p-AKT/t-AKT; **C**, p-mTOR/t-mTOR; **D**, p-ERK/t-ERK; and **E**, p-p38/t-p38. CoQ10: Coenzyme Q10; PI3K: phosphatidylinositol 3 kinase; MAPK: mitogen-activated protein kinase; RANKL: receptor activator of NF‐κB ligand. The data are reported as means±SD. *P<0.05, **P<0.01, ***P<0.001 compared to the control (RANKL), unless otherwise indicated; ANOVA; ns: not significant.

The PI3K/AKT/mTOR and MAPK pathways contribute to the development of osteoporosis. Consequently, we examined the effects of CoQ10 on these two pathways. As demonstrated in Figure 4A-C, the findings revealed that the relative phosphorylation levels of PI3K, AKT, and mTOR were significantly lower in the RANKL + CoQ10 group than in the RANKL group (all P<0.05). However, insignificant differences were found in the relative phosphorylation levels of PI3K, AKT, and mTOR between the RANKL and RANKL + H_2_O_2_ groups. Interestingly, the relative levels of phosphorylated PI3K (P<0.001), total AKT (P<0.01), and total mTOR (P<0.01) were considerably lower in the RANKL + H_2_O_2_ + CoQ10 group than in the RANKL + H_2_O_2_ group. Similarly, compared with those in the RANKL group, the relative phosphorylation levels of ERK and p38 in the RANKL + CoQ10 group were considerably lower but were significantly greater in the RANKL + H_2_O_2_ group (P<0.01 or 0.001). Similarly, the relative levels of phosphorylated ERK (P<0.001) and p38 (P<0.001) were significantly lower in the RANKL + H_2_O_2_ + CoQ10 group than in the RANKL + H_2_O_2_ group (Figure 4D and E). These data indicated that CoQ10 could inactivate the PI3K/AKT/mTOR and MAPK pathways in RAW264.7 cells administered RANKL, regardless of the presence of oxidative stress.

### CoQ10 promoted autophagy via inactivation of the PI3K/AKT/mTOR and MAPK pathways

**Figure 5:**
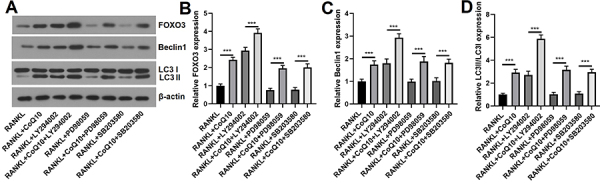
CoQ10 promotes autophagy in RANKL-treated RAW264.7 cells by inactivating the PI3K/AKT/mTOR and MAPK pathways. RAW264.7 cells were pretreated with the PI3K inhibitor LY294002, the ERK inhibitor PD98059, and the p38MAPK inhibitor SB203580, and then, the cells were treated with or without 10^-3^ M CoQ10 in the presence of 50 ng/mL RANKL. The levels of autophagy-related proteins were measured. **A**, Images of autophagy-related proteins determined by western blot; **B**, Quantitative analysis of FOXO3; **C**, Quantitative analysis of Beclin1; **D**, Quantitative analysis of LC3II/LC3I. CoQ10: Coenzyme Q10; RANKL: receptor activator of NF‐κB ligand; FOXO3: forkhead box protein O3. The data are reported as means±SD. ***P<0.001 compared to the corresponding groups; ANOVA.

The PI3K/AKT/mTOR and MAPK pathways play essential roles in the autophagy process. Hence, we explored whether the effects of CoQ10 on autophagy occurred through the regulation of these two pathways. After pretreatment with a PI3K inhibitor (LY294002), an ERK inhibitor (PD98059), or a p38MAPK inhibitor (SB203580), CoQ10 was administered, and the relative levels of FOXO3, Beclin1, and LC3II/LC3I were subsequently measured. As reported in Figure 5A-D, the relative levels of FOXO3, Beclin1, and LC3II/LC3I were significantly greater in the RANKL + CoQ10 + LY294002 group than in the RANKL + LY294002 group (all P<0.001), Moreover, they were greater in the RANKL + CoQ10 + PD98059 group than in the RANKL + PD98059 group, and they were greater in the RANKL + CoQ10 + SB203580 group than in the RANKL + SB203580 group. These data suggested that CoQ10 promoted autophagy via inactivation of the PI3K/AKT/mTOR and MAPK pathways in RAW264.7 cells administered RANKL.

### CoQ10 prevented RANKL-induced osteoclastogenesis via inactivation of the PI3K/AKT/mTOR and MAPK pathways

**Figure 6:**
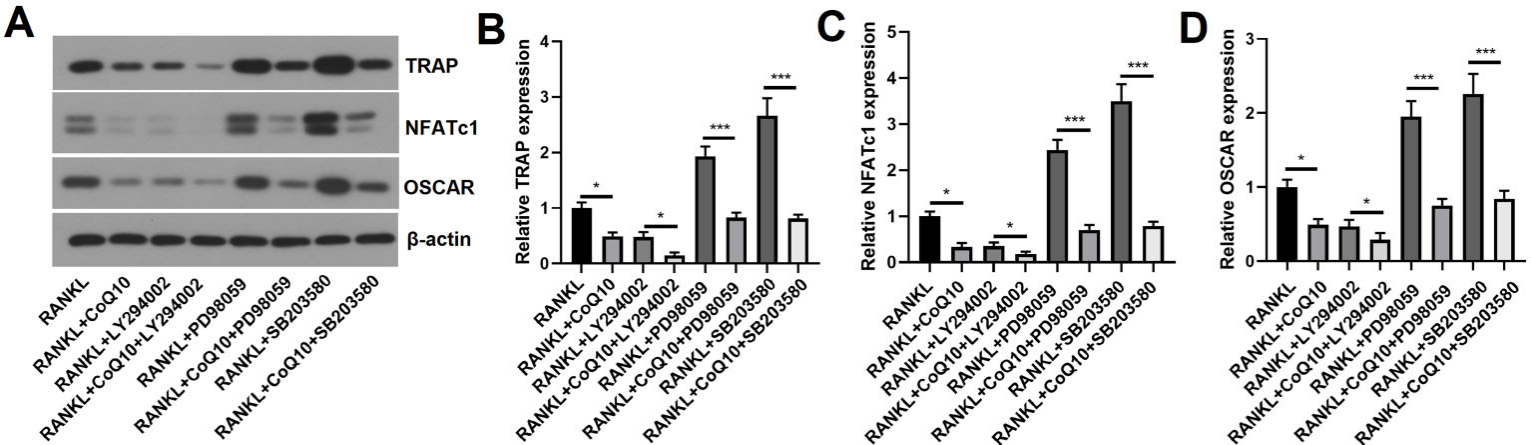
CoQ10 inhibits RANKL-induced osteoclastogenesis by inactivating the PI3K/AKT/mTOR and MAPK pathways in RAW264.7 cells. RAW264.7 cells were pretreated with the PI3K inhibitor LY294002, the ERK inhibitor PD98059, and the p38MAPK inhibitor SB203580, and then the cells were treated with or without 10^-3^ M CoQ10 in the presence of 50 ng/mL RANKL. The protein levels of osteoclast markers were measured. **A**, Images of osteoclast markers determined by western blot; **B**, Quantitative analysis of TRAP; **C**, Quantitative analysis of NFATc1; **D**, Quantitative analysis of OSCAR. CoQ10: Coenzyme Q10; RANKL: receptor activator of NF‐κB ligand; TRAP: tartrate-resistant acid phosphatase; NFATc1: nuclear factor of activated T cells; OSCAR: osteoclast-associated immunoglobulin-like receptor. The data are reported as means±SD. *P<0.05, ***P<0.001 compared to the corresponding groups; ANOVA.

Furthermore, we investigated the impact of CoQ10 on RANKL-induced osteoclastogenesis via the PI3K/AKT/mTOR and MAPK pathways. We observed that the relative levels of osteoclast markers, including TRAP (P<0.05), NFATc1, and OSCAR (all P<0.05), were strongly reduced by administration of CoQ10. After pretreatment with the inhibitors, the relative levels of TRAP, NFATc1, and OSCAR (P<0.05 or 0.001) were considerably lower than those in the corresponding groups (Figure 6A-D). This evidence indicated that CoQ10 prevented RANKL-induced osteoclastogenesis via inactivation of the PI3K/AKT/mTOR and MAPK pathways.

### CoQ10 prevented RANKL-induced osteoclastogenesis via the promotion of autophagy

**Figure 7:**
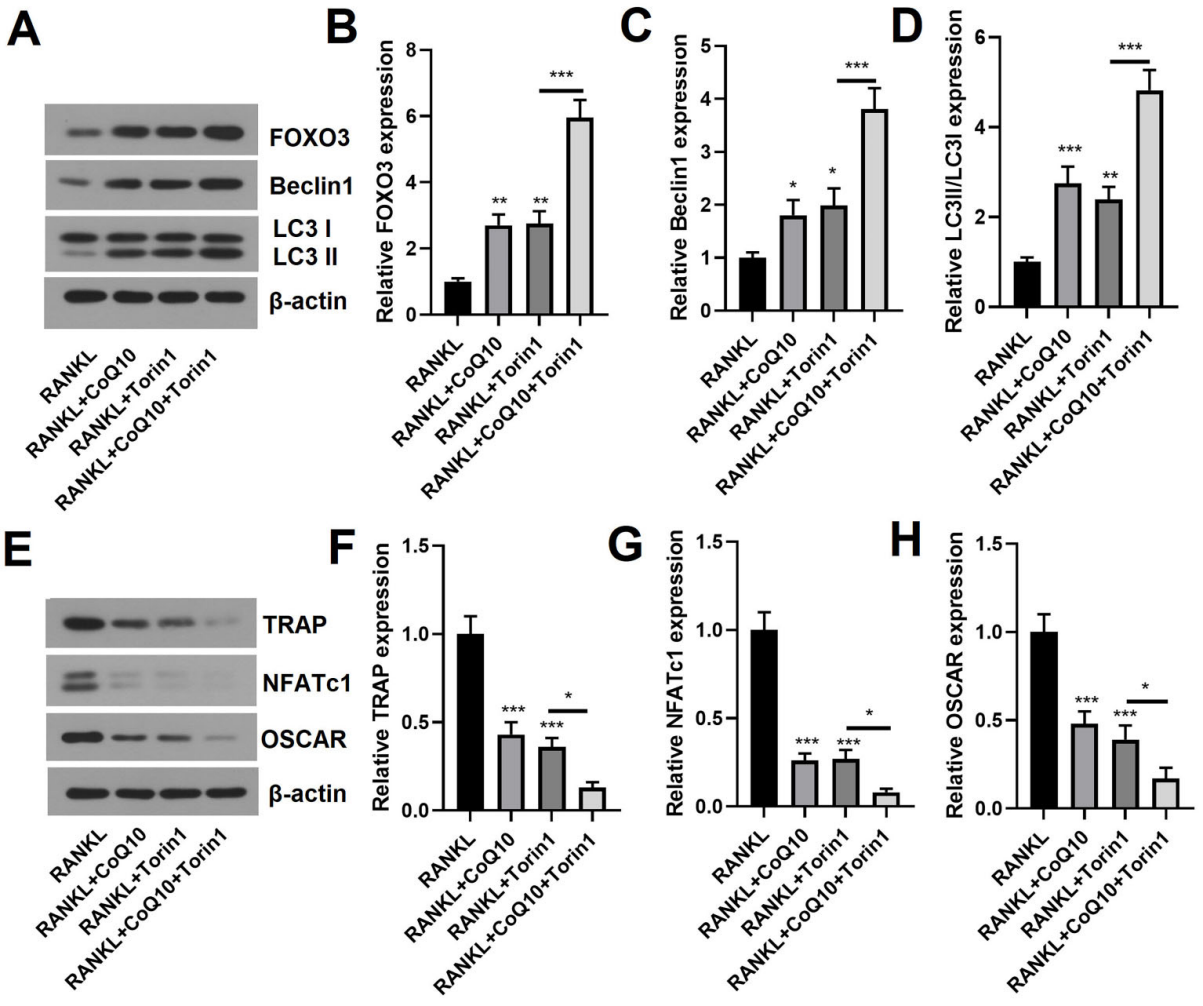
CoQ10 inhibits RANKL-induced osteoclastogenesis via the promotion of autophagy in RAW264.7 cells. RAW264.7 cells were pretreated with the mitophagy agonist Torin1, and then the cells were treated with or without 10^-3^ M CoQ10 in the presence of 50 ng/mL RANKL. The levels of autophagy-related proteins and osteoclast markers were measured. **A**, Images of autophagy-related proteins determined by western blot; **B**, Quantitative analysis of FOXO3; **C**, Quantitative analysis of Beclin1; **D**, Quantitative analysis of LC3II/LC3I; **E**, Images of osteoclast markers determined by Western blot; **F**, Quantitative analysis of TRAP; **G**, Quantitative analysis of NFATc1; **H**, Quantitative analysis of OSCAR. CoQ10: Coenzyme Q10; RANKL: receptor activator of NF‐κB ligand; FOXO3: forkhead box protein O3; TRAP: tartrate-resistant acid phosphatase; NFATc1: nuclear factor of activated T cells; OSCAR: osteoclast-associated immunoglobulin-like receptor. The data are reported as means±SD. *P<0.05, **P<0.01, ***P<0.001 compared to the control (RANKL), unless otherwise indicated; ANOVA.

Finally, we explored the impact of CoQ10 on RANKL-induced osteoclastogenesis via the promotion of autophagy. After pretreatment with the mitophagy agonist Torin1, the relative levels of autophagy-related proteins and osteoclast markers were determined. As shown in Figure 7A-D, the expression of FOXO3 and Beclin1 and the expression of LC3II/LC3I (P<0.05 or P<0.01) were significantly increased by the administration of Torin1. Interestingly, the expression of these genes was significantly increased further by cotreatment with CoQ10 and Torin1 (all P<0.001). Moreover, the data revealed that the relative levels of TRAP, NFATc1, and OSCAR (all P<0.001) were significantly decreased by administration of Torin1. Notably, the expression of these genes was significantly reduced further by cotreatment with CoQ10 and Torin1 (all P<0.05; Figure 7E-H). These findings suggested that CoQ10 prevents RANKL-induced osteoclastogenesis via the promotion of autophagy in RAW264.7 cells.

## Discussion

The purpose of the present study was to investigate the functions of CoQ10 in autophagy during osteoclastogenesis and the potential signaling pathways involved. Our data suggested that CoQ10 prevented RANKL-induced osteoclastogenesis by increasing autophagy via inactivation of the PI3K/AKT/mTOR and MAPK pathways in RAW264.7 cells.

A major cause of fractures is osteoporosis, a disease marked by a decrease in the density of bones and deterioration of bone microarchitecture. According to the current understanding of osteoporosis etiology, osteocyte homeostasis, including differentiation, inflammation, and stress responses, is essential for maintaining cellular function and maintaining bone mass, which are strictly regulated by autophagy (22). It has been reported that autophagy is activated during osteoclast differentiation and can promote RANKL-stimulated osteoclast differentiation (23). The inhibition of autophagy via chloroquine decreases osteoclastogenesis through canonical and noncanonical NF-κB signaling in osteoporosis (24). In addition, specific silencing of Beclin1 in mice damages the functions of osteoclasts, leading to improved cortical bone thickness (23). Moreover, there is evidence that certain drugs can regulate autophagy to modulate osteoclast differentiation. For instance, 1α,25-(OH)_2_D_3_ was reported to increase osteoclastogenesis by enhancing autophagy, while suppressing autophagy via spautin-1 or 3-MA inhibited osteoclastogenesis (25). Therefore, targeting autophagy is considered a potential prevention and treatment option for osteoporosis.

It is well known that CoQ10 plays a biological role as an antioxidant (7,26). An increasing body of data suggests that CoQ10 contributes to the inhibition of osteoclast differentiation by decreasing the expression of genes encoding osteoclast markers, but the exact mechanism is not known. A number of mechanisms have been reported, including a reduction in bone malondialdehyde levels along with an increase in superoxide dismutase levels, regulation of mitochondrial apoptosis, and suppression of ROS production (11,27,28). On the basis of our previous study, CoQ10 may inhibit RANKL-induced osteoclastogenesis by regulating mitochondrial apoptosis and oxidative stress in RAW264.7 cells (11). However, the effects of CoQ10 on autophagy and potential signaling pathways are unclear. Autophagy and apoptosis are interconnected. The two cellular processes share several of the same regulatory signals, and each cellular process can regulate and alter the activity of the other. In addition, numerous studies have shown that oxidative stress can induce autophagy, which can mitigate damage and thus protect cell survival (29). Considering the relationship between autophagy and apoptosis and oxidative stress, we hypothesized that CoQ10 may inhibit RANKL-induced osteoclastogenesis by inducing autophagy. Our study is an in-depth study of previous research, providing different therapeutic mechanisms and molecular basis.

To further explore the potential regulatory mechanism of CoQ10 on osteoclastogenesis, we first treated RAW264.7 cells with 10^-3^ M CoQ10 and performed TRAP staining and MTT. In line with the findings of previous studies (10,11,28), our study showed that CoQ10 significantly decreased the number of TRAP-positive cells but had no obvious toxicity on the cells, suggesting that CoQ10 prevented the osteoclastogenesis induced by RANKL. Thereafter, we tested the expression levels of FOXO3, LC3, and Beclin1 to determine whether CoQ10 inhibited osteoclastogenesis through the regulation of autophagy. The transcription factor FOXO3 plays a significant role in activating genes related to autophagy across a wide range of cell types (30). We found that CoQ10 could meaningfully elevate the expression levels of FOXO3, LC3II, and Beclin1, indicating that CoQ10 promoted autophagy. Our study was similar to previous studies (16-18) in which CoQ10 was shown to play a protective role against different diseases by enhancing autophagy. Interestingly, we also confirmed that CoQ10 further increased autophagy under oxidative stress conditions through treatment with H_2_O_2_. The possible reason might be the powerful antioxidant effect of CoQ10.

Subsequently, we explored the potential signaling pathways involved. The PI3K/AKT/mTOR and MAPK pathways play vital roles in cell growth under both physiological and pathological circumstances (31,32). Numerous studies have revealed that the PI3K/AKT/mTOR and MAPK pathways participate in cell autophagy (33-36). AKT inhibits FOXO3 expression by transferring FOXO3 to the cytoplasm through FOXO3 phosphorylation and inhibiting its entry into the nucleus, thereby inhibiting the expression of autophagy-associated proteins, such as LC3, Beclin1, and ATGs. The MAPK pathway participates in osteoclast differentiation and regulates osteoclast marker secretion (37). ERK and p38 are members of the MAPK family, and ERK is activated by binding of RANK and RANKL, which regulates osteoclast precursor formation (38). The binding of RANK to RANKL allows p38 to be transferred from the cytoplasm to the nucleus and controls osteoclast differentiation (39). The ROS-MAPK pathway is implicated in the apoptotic pathway in RAW264.7 cells, and CoQ10 inhibits ROS production; therefore, CoQ10 likely regulates osteoclast marker production by modulating the ROS-MAPK pathway. Therefore, we hypothesized that the PI3K/AKT/mTOR and MAPK pathways might take part in CoQ10-induced autophagy during osteoclastogenesis. To confirm this hypothesis, we measured the levels of proteins involved in the PI3K/AKT/mTOR pathway and MAPK pathway. As indicated in our results, CoQ10 significantly decreased the levels of p-PI3K, p-AKT, p-mTOR, p-ERK, and p-p38K in both states of stress, suggesting that CoQ10 inactivated these two pathways. To further determine whether the effects of CoQ10 on autophagy occurred through the PI3K/AKT/mTOR and MAPK signaling pathways, we pretreated RAW264.7 cells with inhibitors, including LY294002, PD98059, and SB203580, and then administered CoQ10. The relative expression levels of FOXO3, Beclin1, and LC3II/LC3I were significantly upregulated by these inhibitors, suggesting that CoQ10 promoted autophagy via inactivation of the two signaling pathways in RANKL-treated RAW264.7 cells. Furthermore, the levels of TRAP, NFATc1, and OSCAR were tested. NFATc1 is a downstream target of RANK and a main transcription factor involved in osteoclast differentiation. NFATc1 has been reported to regulate a number of osteoclast-specific genes, such as TRAP and OSCAR (40). Interestingly, the data showed that the protein levels of these genes were significantly decreased by pretreatment with the inhibitors, suggesting that CoQ10 repressed RANKL-induced osteoclastogenesis by activating the PI3K/AKT/mTOR and MAPK pathways. To further confirm these results, we treated cells with the mitophagy agonist Torin1. As expected, the levels of proteins involved in autophagy were upregulated, while the levels of osteoclast markers were downregulated by Torin1. Our results corroborated our suspicions in different directions.

Our study was the first to investigate the effects of CoQ10 on autophagy during RANKL-induced osteoclastogenesis. In addition, we revealed that the PI3K/AKT/mTOR and MAPK pathways contributed to the underlying mechanism. However, this study has several limitations. First, the present investigation involved an *in vitro* experiment; an *in vivo* experiment should be performed to confirm the results. Second, only one concentration of CoQ10 was applied to RAW264.7 cells. Different concentrations need to be explored to reach the optimum dose with no side effects.

**Figure 8:**
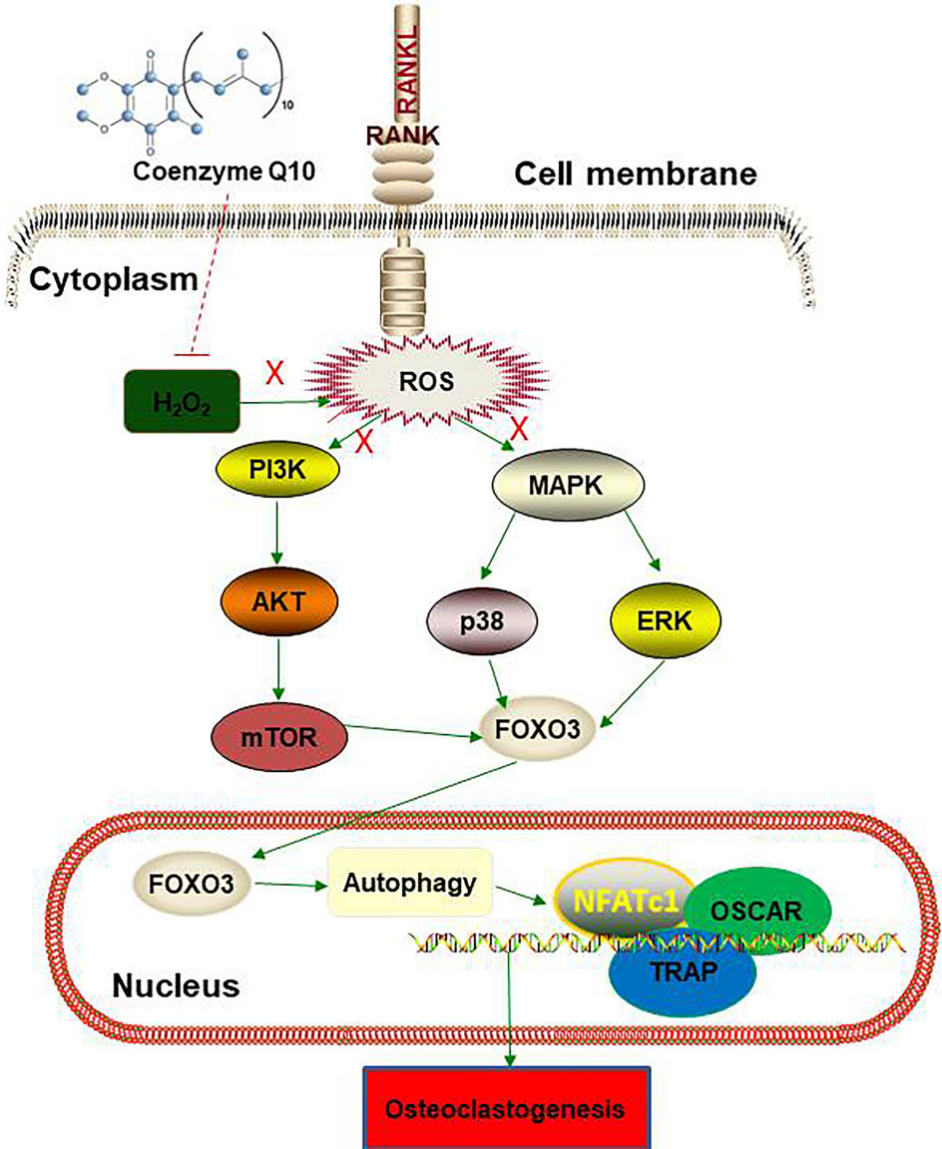
Schematic representation of CoQ10 in RANKL-induced osteoclastogenesis. CoQ10: Coenzyme Q10; RANKL: receptor activator of NF‐κB ligand; ROS: reactive oxygen species; FOXO3: forkhead box protein O3; TRAP, tartrate-resistant acid phosphatase; NFATc1: nuclear factor of activated T cells; OSCAR: osteoclast-associated immunoglobulin-like receptor; PI3K: phosphatidylinositol 3 kinase; MAPK: mitogen-activated protein kinase.

Taken together, our results suggested that CoQ10 prevented RANKL-induced osteoclastogenesis by promoting autophagy via inactivation of the PI3K/AKT/mTOR and MAPK pathways in RAW264.7 cells (Figure 8).
